# Coverage of Recommended Vaccination Among Adult Patients With Asthma in Riyadh, Saudi Arabia

**DOI:** 10.7759/cureus.76620

**Published:** 2024-12-30

**Authors:** Hamdan A Almishrafi, Abdulaziz A Alhaqbani, Waleed I Alshammari, Bader F Alqulaysh, Abdulrahman Y Alnasyan, Saleh A Alwadie, Mohammed A Almozini, Muaath A Alghamdi, Abdullah S Alhqyal, Nawaf Y Alhabi

**Affiliations:** 1 Department of Emergency Medicine, Riyadh Third Health Cluster, Riyadh, SAU; 2 Department of Family and Community Medicine, Riyadh Second Health Cluster, Riyadh, SAU; 3 Department of Family Medicine, Security Forces Medical Center, Riyadh, SAU; 4 Department of Public Health, Shaqra University, Shaqra, SAU

**Keywords:** asthma, high dose corticosteroids, influenza coverage, influenza vaccine, pneumococcal vaccine, seasonal influenza vaccine, vaccination

## Abstract

Introduction

Asthma prevalence among Saudi adults aged 20-44 years in Riyadh is high, with 11.3% reporting physician-diagnosed asthma, exceeding rates in most countries using similar methods. In Aseer province, one out of five adults is estimated to have asthma. Patients with asthma are at higher risk of morbidity and mortality from influenza, pneumococcal, and COVID-19 infections. In Saudi Arabia, the recommended vaccinations for patients with asthma include annual influenza, pneumococcal, and COVID-19 vaccination. Our aim in this study is to estimate the coverage rate of influenza, pneumococcal, and COVID-19 vaccines in patients with asthma who follow up in Riyadh's second health cluster's primary care centers.

Methods

This cross-sectional descriptive study design assessed the coverage rate of recommended vaccinations among patients with asthma. We adhered to the Strengthening the Reporting of Observational Studies in Epidemiology (STROBE) guidelines when reporting the results. Data on patients with asthma was collected from the electronic health records of patients with asthma in Raqeem, a national governmental primary care center electronic health record. Vaccination data were collected from national vaccination records in Seha. Prescribed medications were collected from Wasfaty, a platform for primary care prescriptions.

Results

Overall, 2,689 records of patients with asthma were collected, with 1,441 (53.59%) being males. The median age was 37 years (IQR = 20). Of the patients, 2,273 (84.53%) were Saudi, and 502 (18.67%) received the 2023-2024 influenza vaccine. Only seven (0.26%) patients received the pneumococcal vaccine, while 2,502 (93.05%) patients received any of the COVID-19 vaccines. Patients vaccinated for influenza were significantly older than unvaccinated patients (p < 0.05). Patients prescribed high-dose corticosteroids in the six months prior to the start of the season were significantly more likely to be vaccinated than patients without prescription (p < 0.05).

Conclusion

This study underscores systemic barriers to achieving optimal immunization rates and highlights significant gaps in understanding among patients and healthcare providers. These findings emphasize the need for targeted public health measures, including improved documentation, enhanced education, and stronger recommendations from healthcare professionals during routine asthma management visits. Coordinated efforts by healthcare institutions, such as integrating reminders into electronic health systems, public health initiatives, and further research on vaccination challenges, are vital to increasing vaccination rates in this vulnerable population.

## Introduction

Asthma prevalence among Saudi adults aged 20-44 years in the city of Riyadh is high, with 11.3% reporting physician-diagnosed asthma, exceeding rates in most countries using similar methods [1]. In Aseer province, one out of five adults is estimated to have asthma [2]. In Saudi Arabia, the recommended vaccinations for patients with asthma include annual influenza, pneumococcal, and COVID-19 vaccination [3]. Patients with asthma are at higher risk of morbidity and mortality from influenza, pneumococcal, and COVID-19 infections [4,5]. In the United States of America (USA), respiratory viral infections were linked to 55% of asthma exacerbations treated in emergency rooms [6]. Influenza vaccines reduce complications, hospitalizations, and mortality in patients with asthma and are safe and cost-effective [7,8]. Patients of all ages with high-risk medical conditions benefit from annual influenza vaccination [9]. However, global studies reveal suboptimal vaccination rates among patients with asthma because of factors related to healthcare workers or patients [7,10]. Multiple studies also report fewer complications and hospitalizations after vaccination [7,8]. Pneumococcal pneumonia is a common complication in patients with asthma [11], who face twice the risk of severe pneumococcal disease compared to non-asthmatic patients [12,13]. Physician recommendations are the primary reason for pneumococcal vaccination [14]. A 2023 study at King Saud Medical City in Riyadh, Saudi Arabia, found that healthcare providers failed to recommend pneumococcal or influenza vaccines within six months of discharge [15]. Our aim in this study is to estimate the coverage rate of influenza, pneumococcal, and COVID-19 vaccines in patients with asthma who follow up in Riyadh's second health cluster's primary care centers.

## Materials and methods

Study design

This cross-sectional descriptive study design assessed the coverage rate of recommended vaccinations among patients with asthma. We adhered to the Strengthening the Reporting of Observational Studies in Epidemiology (STROBE) guidelines when reporting the results.

Study setting and population

The study involved patients with asthma visiting primary care centers of the second health cluster in Riyadh, Saudi Arabia.

Inclusion criteria

Patients with asthma aged ≥18 years who visited the primary care centers of the second health cluster in Riyadh, Saudi Arabia, in the past year were included in this study.

Exclusion criteria

Pediatric patients under 18 years were excluded to focus on adults, who are more likely to make independent vaccination decisions.

Sample size estimation

With an asthma prevalence of 11.3% in Riyadh [1] and a population of seven million [16], the estimated number of patients with asthma is approximately 791,000. A minimum of 384 patients is required to achieve a 95% confidence level with a 5% margin of error.

Sampling technique

All adult patients with asthma who visited primary care centers of the second health cluster in Riyadh, Saudi Arabia, between October 2023 and October 2024 were included.

Data collection

Data on patients with asthma were collected from the electronic health records in Raqeem, a national governmental primary care center electronic health record. Vaccination data were collected from national vaccination records in Seha. Prescribed medications were collected from Wasfaty, a platform for primary care prescriptions.

Data management

All data were collected in JavaScript Object Notation (JSON) format from various platforms via the browser.

Statistical analysis

Data were analyzed using R programming language for statistical computing (version 4.3.2; R Foundation for Statistical Computing, Vienna, Austria) [17], along with the tidyverse [18], jsonlite [19], and gtsummary [20] packages.

Patients who received influenza vaccines in Saudi Arabia between September 1, 2023, and August 31, 2024, were labeled as having received the 2023-2024 vaccine. Patients prescribed salmeterol (50 mcg) and fluticasone propionate (250 mcg) inhaler, prednisolone syrup, prednisolone (25 mg) tablets, formoterol fumarate (10 mcg) and fluticasone propionate (250 mcg) inhaler, fluticasone propionate (250 mcg) and salmeterol (25 mcg) inhaler, and vilanterol (25 mcg) and fluticasone furoate (200 mcg) inhaler from March 1, 2023, to September 1, 2023, were considered to have received high-dose corticosteroids. These medications are indicated for a more severe stage of asthma.

Descriptive analysis was performed and presented as demographic data. Continuous variables are presented as medians and interquartile ranges (IQRs), while categorical and ordinal variables are presented as percentages. Categorical variables for vaccinated and non-vaccinated patients were compared using the chi-square test if both groups exceeded 5, and Fisher's exact test if one group had 5 or fewer. Age was compared between groups using the Mann-Whitney U test because of its non-parametric distribution.

Ethical considerations

Ethical approval was obtained from the institutional review board at King Fahad Medical City, Second Health Cluster, Riyadh (approval number: 24-472C).

## Results

Overall, 2,689 records of patients with asthma were collected, with 1,441 (53.59%) being males. The median age was 37 years (IQR = 20). Of the patients, 2,273 (84.53%) were Saudi, and 502 (18.67%) received the 2023-2024 influenza vaccine. Table 1 summarizes the frequencies of prescribed asthma medications during the last six months before the start of the season.

**Table 1:** Frequencies of prescribed asthma medications during the last six months before the start of the 2023-2024 influenza season.

Medication	N = 2,689
Fluticasone propionate (125 mcg) and salmeterol (25 mcg) inhaler	125 (4.6%)
Salmeterol (50 mcg) and fluticasone propionate (250 mcg) inhaler	130 (4.8%)
Salbutamol nebulizer solution	57 (2.1%)
Budesonide (160 mcg) and formoterol fumarate (4.5 mcg) inhaler	352 (13%)
Salbutamol (100 mcg) inhaler	483 (18%)
Budesonide nebulizer solution	52 (1.9%)
Prednisolone syrup	44 (1.6%)
Formoterol fumarate (10 mcg) and fluticasone propionate (250 mcg) inhaler	5 (0.2%)
Fluticasone propionate (125 mcg) inhaler	17 (0.6%)
Prednisolone (5 mg) tablets	68 (2.5%)
Fluticasone propionate (50 mcg) and salmeterol (25 mcg) inhaler	7 (0.3%)
Montelukast (5 mg) tablets	12 (0.4%)
Fluticasone propionate (50 mcg) inhaler	1 (<0.1%)
Budesonide (200 mcg) inhaler	6 (0.2%)
Fluticasone propionate (250 mcg) and salmeterol (25 mcg) inhaler	3 (0.1%)
Prednisolone (25 mg) tablets	2 (<0.1%)
Vilanterol (25 mcg) and fluticasone furoate (200 mcg) inhaler	1 (<0.1%)

**Figure 1:**
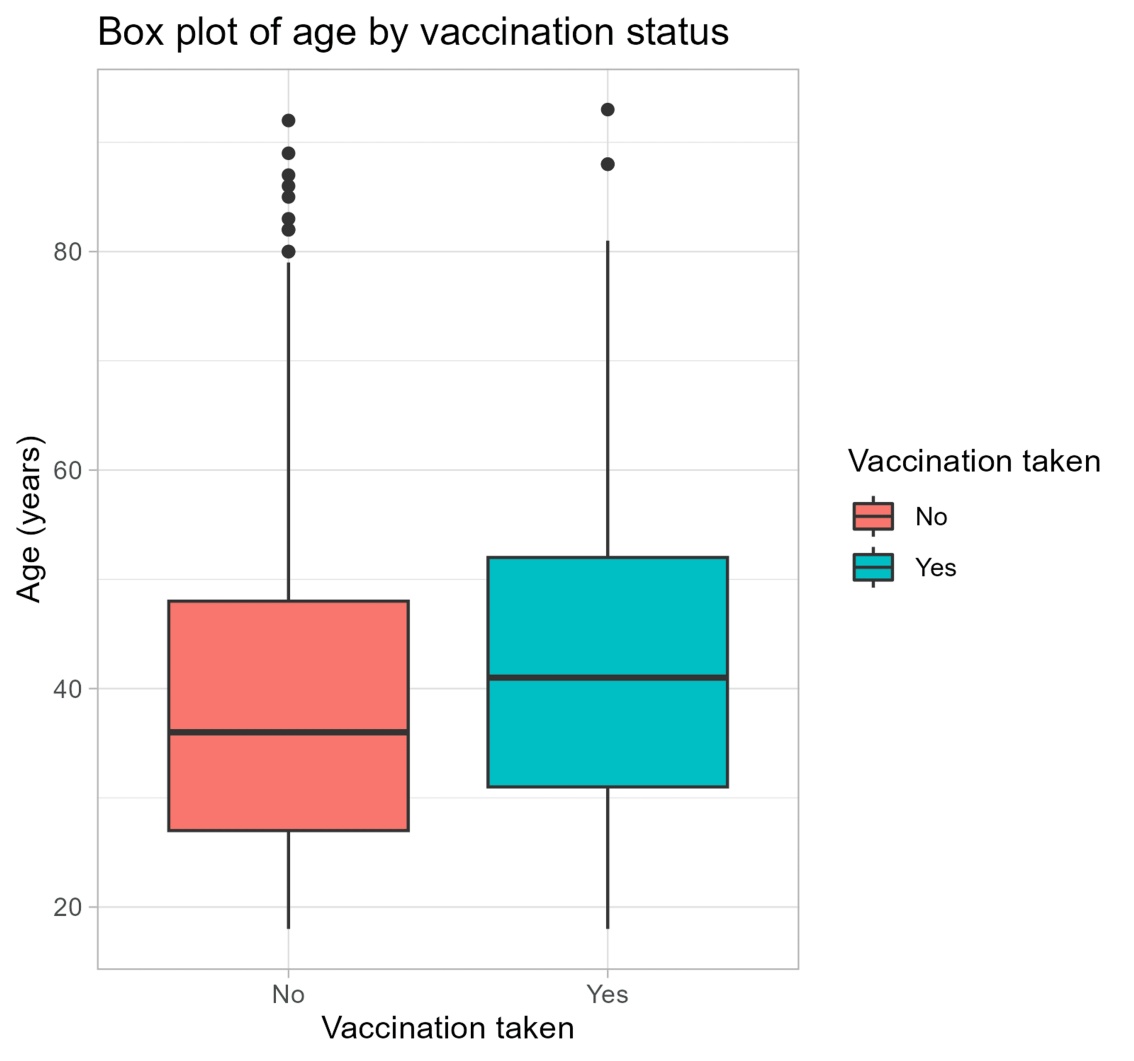
Age distribution by vaccination status.

Only seven (0.26%) patients received the pneumococcal vaccine, while 2,502 (93.05%) patients received any of the COVID-19 vaccines. Patients vaccinated for influenza were significantly older than unvaccinated patients (p < 0.05). Figure 1 shows the age distribution by vaccination status.

Patients aged 50-64 years were more likely to receive the influenza vaccine than older or younger patients (p < 0.05). No significant sex difference was observed in vaccination status (p = 0.13). Similarly, no difference was found between vaccinated and non-vaccinated Saudi and non-Saudi patients (p = 0.8). Patients prescribed high-dose corticosteroids in the six months prior to the start of the season were significantly more likely to be vaccinated than patients without a prescription (p < 0.05). Table 2 summarizes the frequencies and percentages of each variable by vaccination status.

**Table 2:** Frequencies and percentages of each variable by influenza vaccination status. * Wilcoxon rank sum test; # Pearson’s chi-squared test; § Fisher’s exact test A p-value <0.05 was considered statistically significant. Five patients with unknown sex and seven patients with unknown nationality were excluded.

Variable	Influenza vaccination taken		p-value
	No, N = 2,179	Yes, N = 498	
Age: Median (IQR)	36 (27, 48)	41 (31, 52)	<0.001*
Age group (years)			0.002^#^
18–49	1,698 (83%)	354 (17%)	
50–64	378 (76%)	120 (24%)	
≥65	103 (81%)	24 (19%)	
Sex			0.13^#^
Female	1,023 (83%)	215 (17%)	
Male	1,156 (80%)	283 (20%)	
Saudi			0.8^#^
No	327 (81%)	77 (19%)	
Yes	1,852 (81%)	421 (19%)	
Pneumococcal vaccine taken	3 (43%)	4 (57%)	0.026^§^
COVID-19 vaccine taken	2,003 (80%)	488 (20%)	<0.001^#^
Received high-dose corticosteroids	118 (69%)	54 (31%)	<0.001^#^
Fluticasone propionate (125 mcg) and salmeterol (25 mcg) inhaler	92 (75%)	31 (25%)	0.054^#^
Salmeterol (50 mcg) and fluticasone propionate (250 mcg) inhaler	91 (71%)	37 (29%)	0.002^#^
Budesonide (160 mcg) and formoterol fumarate (4.5 mcg) inhaler	250 (71%)	101 (29%)	<0.001^#^
Budesonide nebulizer solution	37 (74%)	13 (26%)	0.2^#^
Salbutamol nebulizer solution	43 (77%)	13 (23%)	0.4^#^
Salbutamol (100 mcg) inhaler	361 (75%)	119 (25%)	<0.001^#^
Formoterol fumarate (10 mcg) and fluticasone propionate (250 mcg) inhaler	4 (80%)	1 (20%)	>0.9^§^
Fluticasone propionate (125 mcg) inhaler	15 (88%)	2 (12%)	0.8^§^
Prednisolone (5 mg) tablets	45 (66%)	23 (34%)	0.001^#^
Fluticasone propionate (50 mcg) and salmeterol (25 mcg) inhaler	6 (86%)	1 (14%)	>0.9^§^
Prednisolone syrup	25 (57%)	19 (43%)	<0.001^#^
Budesonide (200 mcg) inhaler	6 (100%)	0 (0%)	0.6^§^
Fluticasone propionate (50 mcg) inhaler	0 (0%)	1 (100%)	0.2^§^
Montelukast (5 mg) tablets	9 (75%)	3 (25%)	0.5^§^
Prednisolone (25 mg) tablets	1 (50%)	1 (50%)	0.3^§^
Fluticasone propionate (250 mcg) and salmeterol (25 mcg) inhaler	3 (100%)	0 (0%)	>0.9^§^
Vilanterol (25 mcg) and fluticasone furoate (200 mcg) inhaler	1 (100%)	0 (0%)	>0.9^§^

## Discussion

This study found that only one in five patients with asthma received the influenza vaccine for the 2023-2024 season. This rate is lower than that in a 2013 study in Spain, which reported a 35.2% vaccination rate among patients with asthma [7]. A study in the USA (1999-2001) reported vaccination rates of 35.1% in 1999, 36.7% in 2000, and 33.3% in 2001 [5]. Similarly, a 2024 study in Oman reported a higher vaccination rate of 43.6% [21]. These findings highlight a continued failure to meet vaccination recommendations for patients with asthma [3]. Although we expected patients aged 65 years or older to have higher vaccination rates, our data showed that patients aged 50-64 years were more likely to be vaccinated against influenza than older or younger patients, possibly because of greater health awareness. Notably, working-age patients (18-49 years) had vaccination rates lower than the overall average and lower than their US counterparts from previous studies [5]. Patients aged 65 years or older had a very low vaccination rate (19%) compared to the rate (51.9%) reported in a 2017 study in Spain involving the same age group [22]. Vaccination coverage was also lower than that reported in a 2010 study in South Korea, where 81.5% of adults with asthma aged 65 years or older were vaccinated [23]. Approximately one in three patients prescribed high-dose corticosteroid was vaccinated against influenza, a higher rate than the overall vaccination coverage among all patients with asthma. Only seven patients in our study received the pneumococcal vaccine, a very low number, potentially because of several factors. First, the vaccine may not be offered to patients with asthma as recommended [3]. Second, there may be low awareness among patients and healthcare workers. Alternatively, the vaccine may be administered but not documented in the national vaccination record in Seha. Our vaccination rate was much lower than that reported in a 2020 study in Poland, which reported a 7% vaccination rate among patients with asthma [14]. The rate was also lower than that in a 2020 study in Germany, where 7.4% of patients with asthma received both PCV13 and PPSV23, while 22.1% received PPSV23 alone [24]. It was significantly lower than that in a 2017 study in the USA, which reported that 53.7% of working-age patients (18-64 years) with work-related asthma were vaccinated [25]. Given the increased risk of morbidity and mortality from influenza and pneumococcal infections [4,5], we encourage health institutions in Saudi Arabia to ensure these patients receive the recommended vaccines. We also encourage healthcare providers to educate these patients about vaccination guidelines during clinical visits or through public awareness campaigns.

Study limitations

This study relied on electronic health records and the national vaccination registry in Seha, which may not fully capture all vaccinations owing to potential documentation gaps. Future studies should gather data directly from patients using validated questionnaires to minimize the risk of missing or inaccurate entries.

## Conclusions

This study underscores systemic barriers to achieving optimal immunization rates and highlights significant gaps in understanding among patients and healthcare providers. The findings emphasize the need for targeted public health measures, including improved documentation, enhanced education, and stronger recommendations from healthcare professionals during routine asthma management visits. Coordinated efforts by healthcare institutions, such as integrating reminders into electronic health systems, public health initiatives, and further research on vaccination challenges, are vital to increasing vaccination rates in this vulnerable population.

## References

[1] Al Ghobain MO, Algazlan SS, Oreibi TM (2018). Asthma prevalence among adults in Saudi Arabia. Saudi Med J.

[2] Al Ghamdi BR, Koshak EA, Ageely HM, Omer FM, Awadalla NJ, Mahfouz AA (2019). Prevalence and factors associated with adult bronchial asthma in Aseer region, southwestern Saudi Arabia. Ann Thorac Med.

[3] Al-Moamary MS, Alhaider SA, Allehebi R (2024). The Saudi initiative for asthma - 2024 update: guidelines for the diagnosis and management of asthma in adults and children. Ann Thorac Med.

[4] Pesek R, Lockey R (2011). Vaccination of adults with asthma and COPD. Allergy.

[5] Ford ES, Mannino DM, Williams SG (2003). Asthma and influenza vaccination: findings from the 1999-2001 national health interview surveys. Chest.

[6] Atmar RL, Guy E, Guntupalli KK, Zimmerman JL, Bandi VD, Baxter BD, Greenberg SB (1998). Respiratory tract viral infections in inner-city asthmatic adults. Arch Intern Med.

[7] Santos-Sancho JM, López-de Andrés A, Jimenez-Trujillo I, Hernández-Barrera V, Carrasco-Garrido P, Astasio-Arbiza P, Jimenez-Garcia R (2013). Adherence and factors associated with influenza vaccination among subjects with asthma in Spain. Infection.

[8] Vasileiou E, Sheikh A, Butler C (2017). Effectiveness of influenza vaccines in asthma: a systematic review and meta-analysis. Clin Infect Dis.

[9] Hak E, Buskens E, van Essen GA (2005). Clinical effectiveness of influenza vaccination in persons younger than 65 years with high-risk medical conditions: the PRISMA study. Arch Intern Med.

[10] Kaya A, Altınel N, Karakaya G, Çetinkaya F (2017). Knowledge and attitudes among patients with asthma and parents and physicians towards influenza vaccination. Allergol Immunopathol (Madr).

[11] Mirsaeidi M, Ebrahimi G, Allen MB, Aliberti S (2014). Pneumococcal vaccine and patients with pulmonary diseases. Am J Med.

[12] Juhn YJ, Kita H, Yawn BP (2008). Increased risk of serious pneumococcal disease in patients with asthma. J Allergy Clin Immunol.

[13] Talbot TR, Hartert TV, Mitchel E (2005). Asthma as a risk factor for invasive pneumococcal disease. N Engl J Med.

[14] Czaicki N, Bigaj J, Zielonka TM (2020). Pneumococcal vaccine in adult asthma patients. Medical and Biomedical Updates. Advances in Experimental Medicine and Biology.

[15] Alshehri A, Ahmed M, Bagazi D, Alghamdi A (2023). Healthcare providers’ adherence to recommended pneumococcal and influenza vaccination in patients discharged with respiratory diseases from general medical wards. Vaccines (Basel).

[16] (2024). Saudi census. https://portal.saudicensus.sa/portal/public/1/15/45.

[17] (2024). The R project for statistical computing. https://www.r-project.org/.

[18] Wickham H, Averick M, Bryan J (2019). Welcome to the tidyverse. J Open Source Softw.

[19] Ooms J (2014). The jsonlite package: a practical and consistent mapping between JSON data and R objects [PREPRINT]. arXiv.

[20] Sjoberg DD, Whiting K, Curry M, Lavery JA, Larmarange J (2021). Reproducible summary tables with the gtsummary package. R J.

[21] Kharusi ZA, Kalbani RA, Al-Hadhrami R (2024). Frequency of asthma exacerbations and upper respiratory tract infections among adults with asthma according to vaccination status: does the annual influenza vaccine have a protective effect?. Sultan Qaboos Univ Med J.

[22] Suárez-Varela MM, Llopis A, Fernandez-Fabrellas E (2018). Asthma and influenza vaccination in elderly hospitalized patients: matched case-control study in Spain. J Asthma.

[23] Chung JH, Kim TH, Han CH (2018). Factors influencing influenza vaccination among South Korean adult asthma patients: a nationwide population-based cross-sectional study. J Asthma.

[24] Mohr A, Plentz A, Sieroslawski A, Pezenburg F, Pfeifer M, Salzberger B, Hitzenbichler F (2020). Use of pneumococcal and influenza vaccine in patients with COPD, asthma bronchiale and interstitial lung diseases in south east Germany. Respir Med.

[25] Dodd KE, Mazurek JM (2017). Pneumococcal vaccination among adults with work-related asthma. Am J Prev Med.

